# When feelings matter: moderating role of affective attitude on cognitive attitude and adolescents’ out-of-school physical activity

**DOI:** 10.3389/fspor.2026.1775703

**Published:** 2026-03-27

**Authors:** Xiaozhen Guo, Yaogang Han, Ying Liu, Pan Li

**Affiliations:** 1 Department of Physical Education, Tongji University, Shanghai, China; 2 School of Physical Education, Shanghai University of Sport, Shanghai, China; 3 School of Psychology, Shanghai University of Sport, Shanghai, China; 4 School of Athletic Performance, Shanghai University of Sport, Shanghai, China

**Keywords:** adolescents, affective attitude, cognitive attitude, moderation, out-of-school physical activity

## Abstract

**Introduction:**

This study tested whether affective attitude toward physical activity (PA) acts as a boundary condition for the cognitive attitude–behavior link in adolescents.

**Methods:**

Participants were 1,849 middle- and high-school students in Shanghai, China (51% male; Mage = 15.12). Affective and cognitive attitudes were assessed with semantic differential scales, and out-of-school PA behavior was assessed 5–7 days later.

**Results:**

Confirmatory factor analysis supported distinct affective and cognitive attitude factors. Moderation analyses indicated a significant Cognitive × Affective interaction (*β* = .48, *p* < .01). Johnson–Neyman probing indicated that the cognitive attitude–PA association strengthened as affective attitude increased; in this sample, the conditional effect became statistically distinguishable from zero (*α* = .05) when affective attitude was in the mid-to- high range (approximately > 4.16 on the 7-point scale) and increased progressively at higher affective levels.

**Discussion:**

These findings extend attitude—behavior models by indicating that affective evaluations condition the strength of the cognitive attitude—behavior association and generate a testable hypothesis for future intervention research—namely, that enhancing positive affective evaluations/enjoyment may increase the effectiveness of cognitive benefit messaging—which should be evaluated in experimental designs.

## Introduction

Attitude has consistently been identified as a strong predictor of physical activity (PA) behavior (1, 2). This is particularly important in adolescence, when insufficient PA remains highly prevalent worldwide (3) despite clear public health recommendations (4). Attitude is frequently targeted in interventions aimed at promoting PA (5, 6). As a psychological construct, attitude comprises cognitive and affective components (7) that align with contemporary affect-focused frameworks highlighting distinct affective vs. reflective pathways to behavior (8). For instance, individuals form attitudes toward PA based on cognitive evaluations of its benefits or harms and affective evaluations related to anticipated enjoyment or displeasure associated with doing PA (9). These components are commonly referred to as cognitive and affective attitudes, respectively (9–11).

Adolescence is a particularly important developmental period for understanding PA determinants because PA levels are low and commonly decline across the teenage years. Globally, approximately 80% of adolescents do not meet recommended PA levels (3), and recent syntheses emphasize adolescence as a period of rapid biological, psychological, and social transition that can disrupt PA participation and complicate intervention efforts (12). Importantly, PA behavior shows low-to-moderate tracking from adolescence into young adulthood (13), suggesting that determinants of PA during adolescence may have lasting implications. As adolescents gain greater autonomy over discretionary time, attitudes may become especially influential for decisions about out-of-school PA, making this developmental period a theoretically and practically important focus for the current study.

Traditionally, cognitive and affective attitudes have been conceptualized as independent predictors of PA behaviors (10, 14, 15). However, contemporary affective science and dual-process perspectives suggest that affective valuation can shape when reflective, instrumental beliefs translate into action (8, 16, 17). Consistent with this view, emerging empirical work has begun to integrate affective and instrumental attitude processes in predicting PA (18) and suggests that affective attitude may moderate the effects of cognitive attitude on PA behavior (19–21). To further understand this interaction, the purpose of the current study was to examine the extent to which affective attitude moderates the relationship between cognitive attitude and out-of-school PA behavior among adolescents.

Recent theoretical syntheses argue that affect-related constructs (e.g., affective attitudes, anticipated affect, affective responses, and implicit affective associations) play a central role in PA decision making and may help explain why cognitively endorsed benefits often yield limited behavior change (8). The Affective–Reflective Theory (ART) proposes that exercise-related cues elicit automatic affective valuations that can facilitate or inhibit action, with reflective evaluations exerting greater influence when they are supported by positive affective valuations (16). Similarly, the Theory of Effort Minimization in Physical Activity (TEMPA) highlights an automatic attraction to lower-effort options that can undermine reflective intentions, particularly under fatigue or cognitive load (17). These perspectives converge on the prediction that affective evaluations may function as a boundary condition for the impact of instrumental/cognitive evaluations on PA—motivating the moderation hypothesis tested in the present study.

Cognitive attitude reflects an individual's overall evaluation based on beliefs about the attributes of a behavior (7). For example, some individuals perceive PA as beneficial to health and fitness, whereas others may view it as a waste of time or risky due to potential injuries. Such beliefs form the basis of cognitive attitudes toward PA. In contrast, affective attitude pertains to the overall evaluation based on feelings and emotions associated with a behavior (7). Individuals who find PA enjoyable and energizing develop positive affective attitudes, whereas those experiencing boredom or discomfort develop negative affective attitudes. Importantly, affective attitude should be distinguished from affective responses during PA. Affective responses refer to the momentary feelings (e.g., pleasure–displeasure, positive/negative affect) experienced while engaging in PA, whereas affective attitude reflects a generalized evaluative stance toward PA that is shaped by remembered and anticipated affective experiences across repeated occasions (8, 22). Thus, although affective responses and affective attitudes are related, they are conceptually distinct constructs that operate at different levels of analysis.

The distinction between cognitive and affective attitudes has been supported across various domains such as blood donation, abortion, college examinations (23–25), and health behaviors like smoking, flossing, and vitamin use (26). Although cognitive and affective attitudes toward PA are correlated [r = .49–.77, (9)], numerous studies have validated their conceptual separation within the PA context (9, 10, 15). Both cognitive and affective attitudes independently demonstrate positive associations with PA behaviors (10, 11, 15). However, emerging research suggests this assumption of independence may not be adequate, as affective attitudes could moderate the influence of cognitive attitudes on PA behavior.

One influential line of work focuses on affective responses during PA (i.e., the acute affective experience while being active). This work shows that more positive in-task affective responses are associated with higher subsequent PA participation (19, 27), and it has been argued that individuals may remain inactive despite recognizing PA benefits if PA is repeatedly experienced as unpleasant (19, 28). Conceptually, repeated affective responses may contribute to the development of more positive or negative affective attitudes over time (22). Accordingly, the affective-response literature provides a plausible mechanism for why affective attitude may function as a boundary condition that determines when cognitive benefit beliefs translate into behavior.

In adolescents, ambulatory assessment research suggests PA is often followed by increases in positive affect and energy, although findings are mixed and likely context-dependent (29). Moreover, recent youth-focused scholarship increasingly emphasizes enjoyment as a key affective determinant and intervention target (30, 31), including pedagogical work highlighting strategies that support positive affective learning outcomes in school physical education (53). Although Ekkekakis et al. primarily examined during-behavior affect, research suggests affective attitudes reflect individuals' past emotional experiences with PA (22), supporting the plausibility that affective attitude moderates cognitive attitude's influence on PA. Empirical studies examining this moderation effect, however, have yielded mixed findings. Kiviniemi, Voss-Humke, and Seifert (32) explicitly tested affective attitude's moderation of cognitive attitude and PA among adults, finding no significant effects, potentially due to limited sample size (*n* = 433). In contrast, Lawton et al. (26), examining affective-cognitive attitude divergence, found significant interaction effects suggesting moderation between affective and cognitive attitudes on PA behavior. To resolve these inconsistencies, larger sample sizes are needed due to statistical power concerns in interaction analyses (33).

Although the possibility that affective evaluations may condition the effects of cognitive evaluations has been suggested [e.g., (19, 26)], the empirical contribution of the present study is to clarify and translate this proposition in an adolescent context. Prior work has produced mixed findings and has often emphasized whether an interaction exists, with less attention to identifying where (i.e., at what affective-attitude level) cognitive beliefs begin to matter in a way that is actionable for intervention design. The present study contributes by providing (a) a high-powered test of the Cognitive × Affective interaction in a large adolescent sample, (b) evidence in a non-Western youth context focused specifically on out-of-school PA, and (c) an intervention-relevant quantification of the boundary condition using Johnson–Neyman analysis to identify the affective-attitude value above which cognitive attitude becomes predictive of behavior. The objective of this study was to test a moderation (boundary-condition) framework in which adolescents’ affective attitude toward physical activity conditions the extent to which cognitive attitude predicts subsequent out-of-school physical activity behavior. From an applied perspective, this moderation test was intended to clarify whether cognitive “benefit” beliefs are more strongly associated with behavior at higher levels of positive affective evaluation (enjoyment/pleasantness), thereby generating hypotheses about intervention targeting and sequencing that should be tested in future longitudinal and experimental research.

Although we posit a moderation (boundary-condition) hypothesis, several competing explanations are plausible and should be distinguished. First, an additive/independent-effects account—common in expectancy–value and planned-behavior traditions—suggests that affective and cognitive attitudes each contribute uniquely to PA, but do not interact; under this view, interventions can raise PA by increasing either component, and the cognitive attitude–behavior association should be similar across affective levels. Second, a mediation account suggests that one component primarily influences behavior through the other (e.g., instrumental beliefs shape feelings about PA over time, or repeated affective experiences shape perceived benefits), implying an indirect pathway rather than moderation. Third, an affective-dominance explanation derived from contemporary affective science proposes that affective valuation is the more proximal driver of action, such that cognitive evaluations add little once affect is considered. In contrast, affective–reflective perspectives (e.g., affective valuation as a “gatekeeper”) imply that instrumental beliefs are most likely to translate into PA when PA is experienced as enjoyable/positive—thereby predicting an interaction. Accordingly, the present study tests both main effects and the Cognitive × Affective interaction to empirically adjudicate between additive and boundary-condition explanations. We hypothesized that the positive association between cognitive attitude and out-of-school PA behavior would be stronger at higher levels of affective attitude, and would be weak or non-significant when affective attitude is low.

## Methods

### Power analysis

The sample size in this study was estimated based on a multiple regression model with two predictors (cognitive attitude and affective attitude) and one outcome variable [leisure-time MVPA (i.e., physical activity outside of school)], since the hierarchical regression and moderation analyses used were analogous to this regression model. Two approaches were employed to estimate the required sample size: one based on the overall model R² and the other based on the semi-partial correlation coefficients for each predictor. The estimates of these coefficients were drawn from existing literature (15, 34–36). These studies were selected because they focused either on Chinese students/adolescents or students from other countries. Several meta-analytical studies were also reviewed to estimate the correlation coefficients of predictors (37–39). To maintain a conservative estimate, the lowest values of R² and correlation coefficients reported in the literature were selected. Specifically, the estimated R² used was.16, and the estimated correlation coefficients for cognitive and affective attitude were.08 and.40, respectively. The required sample size was calculated using a fixed-effects linear multiple regression model in G*Power. To achieve a power of.80 at an alpha level of.05, the estimated sample sizes based on the R² and correlation coefficients were 62, 1,101, and 44, respectively. Therefore, a sample size larger than 1,101 was considered necessary to ensure sufficient statistical power (.80) at a significance level of.05.

### The sample

Participants were recruited from six middle schools and four high schools in Shanghai, China, selected through the researchers' professional networks. A total of 1,849 adolescents participated (1,065 middle-school students, 784 high-school students; 51% male, 49% female; mean age = 15.12 years). This study protocol was reviewed and approved by the Shanghai University of Sport Institutional Review Board (Approval No. 102772022RT045). Because participants were adolescents, written informed consent was obtained from parents/legal guardians and written assent was obtained from students prior to data collection. Participation was voluntary, and responses were collected anonymously.

### Variables and measures

#### Cognitive and affective attitude

Cognitive and affective attitudes were assessed using a series of 7-point bipolar adjective scales, consistent with prior studies [e.g., (9, 24, 40, 41)]. Affective attitude was measured by three pairs of bipolar adjectives: enjoyable–unenjoyable, interesting–boring, and relaxing–stressful, each preceded by the statement, “I feel that doing PA regularly outside of school is…”. Cognitive attitude was measured by three pairs of bipolar adjectives: useful–useless, beneficial–harmful, and wise–foolish, each preceded by the statement, “I think that doing PA regularly outside of school is…”. A pilot study was conducted to determine the reliability and validity of the semantic differential scale. A convenient sample of 67 middle school and 59 high school students completed the scale. Acceptable validity and reliability were found for middle school (construct validity: *χ*² = 20.10, df = 8, *p* < .01; TLI = .95; CFI=.97; SRMR=.04; Cronbach's alpha: cognitive attitude = .89, affective attitude = .97) and high school students (construct validity: *χ*² = 25.65, df = 8, *p* < .01; TLI = .92; CFI=.95; SRMR=.02; Cronbach's alpha: cognitive attitude = .92, affective attitude = .91). These items assess participants' generalized evaluative attitudes/anticipated affect toward doing PA outside of school, rather than momentary affective responses during a specific PA bout.

#### Out-of-school PA

Physical activity (PA) outside of the school was assessed using three items: “In the last 7 days, on how many days right after school, did you do sports, dance, or play games in which you were very active?”, “In the last 7 days, on how many evenings did you do sports, dance, or play games in which you were very active?”, and “On the last weekend, how many times did you do sports, dance, or play games in which you were very active?”. Each item was rated on a 5-point scale. The average score of these three items was calculated to represent students' PA level outside of the school.

#### Translation procedures and trustworthiness

These questionnaire items were independently translated from English to Chinese by two bilingual translators, each holding a doctoral degree and specializing in physical education or exercise psychology. The translation followed the self-report measures translation guidelines (42). First, one translator completed a forward-translation, and the other conducted a backward-translation. The backward-translated version was then compared with the original English version, and any discrepancies were discussed and resolved until both translators reached agreement. Next, to evaluate readability and face validity, six in-service physical education teachers (three middle school and three high school) and ten students (five middle school and five high school) reviewed the Chinese questionnaire. The items were revised according to their feedback; for instance, specific physical activity examples commonly performed by Chinese middle and high school students (e.g., running, jump rope, biking) were included. All reviewers indicated that the final questionnaire was clear and understandable for middle and high school students.

### Data collection

Data collection took place at the beginning of physical education (PE) classes with assistance from PE teachers. Students first completed measures assessing their cognitive and affective attitudes toward physical activity (PA). Approximately five to seven days later, students completed the questionnaire measuring their out-of-school PA behavior. Researchers were present throughout data collection to immediately address any questions from students.

### Data analysis

Confirmatory factor analysis (CFA) was first conducted to evaluate the validity of the two-factor attitude model (cognitive and affective attitudes). Model fit was assessed using several fit indices recommended by Hu and Bentler (43): Chi-square test (*p* > .05), Standardized Root Mean Square Residual (SRMR ≤ .08), Root Mean Square Error of Approximation (RMSEA ≤ .06), and Comparative Fit Index (CFI ≥ .95). The CFA was performed using Mplus 7.4.

To examine the research question concerning the moderating role of affective attitude on the relationship between cognitive attitude and out-of-school physical activity (PA), hierarchical regression analysis was employed. Cognitive and affective attitudes were entered as predictors in the first step, and their interaction term was entered in the second step, with out-of-school PA as the dependent variable. Additionally, moderation analysis using Hayes' PROCESS macro (Model 1) was conducted to further understand the moderation effect. PROCESS provides Johnson-Neyman conditional effect analyses, identifying specific values of affective attitude at which cognitive attitude significantly influences out-of-school PA, thereby offering deeper insight into the moderation effect. Hierarchical regression and moderation analyses were performed using IBM SPSS 28.0. We elected to test moderation using observed scale scores (rather than latent interaction SEM) because this approach allows transparent estimation of conditional effects and a Johnson–Neyman region-of-significance in the original 1–7 response metric, which is central to the applied interpretability of the findings (i.e., identifying the range of affective-attitude values at which cognitive attitude becomes statistically predictive of behavior). Importantly, the attitude measures demonstrated strong psychometric properties (high internal consistency and clear factorial structure), which helps reduce concern that measurement error would materially alter the moderation pattern. Nevertheless, future research could extend the present work using latent-variable interaction models and multi-group approaches after establishing measurement invariance.

## Results

The confirmatory factor analysis results indicated that the two-factor attitude model showed an acceptable fit to the data (*χ*² = 83.43, df = 8, *p* < .01; RMSEA = .05; CFI = .98; SRMR = .03; factor loadings ranged from.85 to.96). Cronbach's alpha values were.90 for cognitive attitude and.93 for affective attitude, suggesting good psychometric properties. Table 1 presents descriptive statistics and bivariate correlations for all variables. Participants reported highly positive cognitive attitudes and moderately positive affective attitudes toward physical activity (PA). Cognitive and affective attitudes were moderately correlated with each other and showed small positive correlations with out-of-school PA. To address the research question, hierarchical regression analysis was conducted, and results are presented in Table 2. The analysis revealed that the interaction term between cognitive and affective attitudes significantly predicted out-of-school PA (*β* = .48, *p* < .01), indicating a significant moderation effect of affective attitude on the relationship between cognitive attitude and out-of-school PA. The moderation analysis using Hayes' PROCESS macro confirmed this interaction effect. Specifically, the Johnson–Neyman conditional effect analysis (Table 3) showed that the conditional effect of cognitive attitude on out-of-school PA increased as affective attitude increased. At lower affective attitude values, this conditional effect was not statistically distinguishable from zero (*p* > .05); in this sample, it reached statistical significance at *α* = .05 when affective attitude exceeded approximately 4.16 on the 7-point scale and continued to strengthen at higher values. This Johnson–Neyman value represents a statistical region-of-significance boundary (dependent on sampling variability and model specification) rather than a discrete psychological cutoff. These findings suggest that cognitive attitude has little impact on adolescents' out-of-school PA when affective attitude is negative or low, but has an increasingly strong positive impact as affective attitude becomes more positive.

**Table 1:** Descriptive statistics and bivariate correlations of the variables.

Variables	Mean/SD	Skewness/Kurtosis	1	2
1 Cognitive attitude	6.32/.96	−1.73/3.44	–	
2 Affective attitude	6.10/1.18	−1.54/2.25	.82**	
3 Out-of-school PA	3.65/1.26	.02/−.31	.36**	.40**

All correlation coefficients are significant at .01 level.

**Table 2:** Hierarchical regression of out-of-school PA on cognitive and affective attitude.

Independent variables	*β*	*R^2^*	*R^2^* change	*F* value
Step 1		.16**	.16**	194.59**
Cognitive attitude	.09*			
Affective attitude	.33**			
Step 2		.17**	.01*	133.54**
Cognitive attitude	−.10			
Affective attitude	.01			
Interaction term	.48**			

*β* values are standardized regression coefficients from the final stage of the regression analysis.

* *p* < .05;

** *p* < .01.

**Table 3:** Johnson-Neyman conditional effect of cognitive attitude on out-of-school PA.

Affective attitude value	Effect	*t*	*p* value
1.00	−.075	−.970	.332
1.60	−.043	−.622	.534
2.20	−.012	−.184	.854
2.80	.021	.364	.716
3.40	.052	1.022	.307
3.70	.068	1.385	.166
4.00	.084	1.760	.079
4.16	.093	1.961	.050
4.30	.100	2.134	.033
4.90	.132	2.824	.005
5.50	.164	3.362	.001
6.10	.196	3.715	.000
6.70	.228	3.912	.000
7.00	.244	3.968	.000

## Discussion

The results of this study supported the hypothesis regarding the moderating role of affective attitude on the relationship between cognitive attitude and out-of-school physical activity (PA). Specifically, affective attitude significantly moderated the relationship between cognitive attitude and out-of-school PA, providing insights into how these two attitudinal components interact to influence PA behavior among adolescents.

One important finding of this study was that cognitive attitude had no significant effect on out-of-school PA when affective attitude was negative. This implies that if adolescents hold negative affective evaluations of PA, their cognitive evaluations—regardless of how positive or beneficial they perceive PA to be—tend not to influence their PA behavior. This finding provides potential insights into previous experimental studies that attempted to enhance PA behaviors by manipulating cognitive and affective attitudes through informational messaging (5, 44, 45). For instance, Conner and colleagues (5) found that providing affective information significantly increased PA, whereas cognitive information alone did not produce similar effects. The results of the current study suggest that the non-significant changes observed in cognitive message groups could be due to participants holding low or negative affective attitudes toward PA.

Additionally, this study demonstrated that cognitive attitude had a progressively stronger influence on out-of-school PA as affective attitude became more positive. This finding indicates that among adolescents who hold positive affective evaluations of PA, cognitive beliefs about PA become increasingly influential. Sirriyeh and colleagues (45) previously reported findings consistent with this idea: among physically active adolescents (who likely possess positive affective attitudes), both cognitive and affective messages similarly improved PA behaviors, whereas among inactive adolescents (likely holding negative affective attitudes), only affective messages produced significant PA improvements. Thus, affective attitude appears to be crucial in determining when cognitive information effectively influences PA behaviors. Future experimental research controlling for participants' baseline affective attitudes is needed to validate this interpretation.

These results also shed light on why many individuals remain physically inactive despite recognizing PA's benefits and importance. Research from Australia, for example, shows that although nearly all adults acknowledge the importance of PA, approximately 85% remain insufficiently active (46, 54). Similarly, other studies indicate weak or non-significant associations between cognitive knowledge of PA and actual PA behavior (47, 48). The current findings suggest that this disconnection might occur because negative affective attitudes toward PA inhibit the influence of cognitive beliefs on actual behavior. Future research that explicitly compares groups with positive and negative affective attitudes would provide deeper insights into these relationships. Importantly, the present study assessed affective attitude (a generalized evaluation/anticipated affect), not in-task affective responses; nevertheless, the results are consistent with the broader proposition that negative affective valuation can blunt the translation of reflective benefit beliefs into PA behavior (19).

This study offers theoretical and applied implications. Theoretically, identifying the Cognitive × Affective interaction suggests that affective and cognitive attitudes may not operate purely additively in predicting PA behavior; future attitude–behavior models may benefit from explicitly considering affective attitude as a boundary condition that shapes when cognitive evaluations are more strongly associated with behavior. Applied implications should be interpreted cautiously because the design is observational (and thus cannot establish causal effects or confirm intervention sequencing). Nevertheless, the pattern observed here generates a testable hypothesis for future intervention research: cognitive benefit messaging may be more strongly associated with behavior among adolescents with more positive affective evaluations of PA, and interventions that increase enjoyment/positive affective evaluations may enhance the behavioral relevance of cognitive beliefs. These hypotheses should be evaluated using longitudinal and experimental designs.

The interaction term accounted for a small incremental proportion of variance (*Δ*R² = .01). This magnitude is common for interaction effects in behavioral research, and the value of moderation is often less about large gains in overall explained variance and more about identifying meaningful heterogeneity in associations across levels of a moderator. Consistent with this, Table 3 shows that the conditional effect of cognitive attitude ranges from near zero at low affective attitude (e.g., effect = −.075 at affective attitude = 1.0) to more substantial positive associations at higher affective attitude (e.g., effect ≈.196–.244 at affective attitude = 6.1–7.0). Thus, while the global increment in R^2^ is modest, the interaction meaningfully changes the slope of the cognitive attitude–PA association across the affective-attitude continuum, which is relevant for theory refinement and for informing targeted hypotheses in future intervention testing.

The observed moderation effects may also be explained by findings on how affect influences cognitive processing. Prior research indicates that negative affect tends to impair executive functions and reduce people's ability to effectively focus on or utilize cognitive information (49, 50). Conversely, positive affect can enhance cognitive processing, making it more flexible, creative, and efficient, which facilitates decision-making and behavior change (51, 52). Adolescents with negative affective attitudes toward PA might struggle to effectively utilize cognitive information about PA benefits, reducing its impact on their behavior. Adolescents with positive affective attitudes, however, may process cognitive information more thoroughly, resulting in stronger associations between cognitive attitudes and out-of-school PA. Collectively, the findings underline the essential role affective attitude plays in determining when cognitive attitudes effectively influence PA behaviors. Future research and intervention design should prioritize affective attitude enhancement to achieve meaningful and sustained improvements in adolescent out-of-school PA behavior.

Several limitations should be acknowledged. First, schools were recruited through the researchers' professional networks, yielding a convenience sample from a single metropolitan area (Shanghai). Although the sample was large and included both middle and high school students, this recruitment approach may limit external validity and the generalizability of the findings to adolescents in other regions or sociocultural contexts. Future studies should replicate these results using more diverse samples and, when feasible, probability-based recruitment strategies. Second, out-of-school PA was assessed exclusively via self-report, which may be affected by recall error and social desirability bias and could lead to misclassification or overestimation of PA. Future research should incorporate objective PA assessment (e.g., accelerometry) and/or repeated, time-sensitive measurement approaches to reduce measurement bias and to test whether the observed moderation pattern holds with objectively measured PA.

The present findings were obtained in a sample of adolescents from Shanghai, China, and cultural context may shape both affective and cognitive evaluations of physical activity. For example, the salience of academic demands, school routines, and socially valued uses of discretionary time may influence how adolescents evaluate out-of-school PA (e.g., as enjoyable/stress-reducing vs. time-competing with study) and how strongly benefit beliefs translate into behavior. Accordingly, the observed moderation pattern—and the specific Johnson–Neyman region-of-significance boundary—should not be assumed to generalize universally across cultural settings. Replication in diverse cultural and socioeconomic contexts (including adolescents in other regions of China and in different countries) is needed to determine the cross-cultural robustness of the affective-attitude boundary-condition pattern.

Additional analytic extensions should also be considered. First, we did not test whether the moderation pattern differs across key subgroups such as gender or school level. Evaluating subgroup robustness is an important next step; however, such comparisons are ideally preceded by tests of measurement invariance to ensure that affective and cognitive attitude constructs are comparable across groups. Second, although we supported the measurement model via CFA, the moderation test was conducted using manifest scale scores. Latent-variable interaction SEM could further reduce measurement error, but it requires additional modeling decisions (e.g., interaction specification and scaling of latent factors) and typically yields effects on a latent metric, which translates conditional effects into intervention-relevant guidance. Future studies should build on the current findings by pairing invariance testing with multi-group moderated models and by examining latent interactions while developing clear procedures to translate conditional effects into intervention-relevant thresholds.

## Conclusions

In conclusion, the present findings suggest that affective attitude functions as a boundary condition for the cognitive attitude–out-of-school PA association among adolescents. Specifically, cognitive attitude was not statistically distinguishable from zero at lower affective attitude values, but became increasingly predictive as affective attitude increased (with Johnson–Neyman analysis indicating significance above approximately 4.16 in this sample). This value should be interpreted as a statistical region-of-significance boundary rather than a universal psychological cutoff. Because the design is observational, the practical implications are hypothesis-generating rather than causal: future research should test—using longitudinal and experimental designs—whether enhancing positive affective evaluations/enjoyment increases the effectiveness of cognitive benefit messaging and supports sustained PA behavior.

## Data Availability

The data that supports the findings of this study are available from the corresponding author upon reasonable request.
